# Development and validation of an educational booklet for healthy eating during pregnancy^1^

**DOI:** 10.1590/0104-1169.3313.2459

**Published:** 2014

**Authors:** Sheyla Costa de Oliveira, Marcos Venícios de Oliveira Lopes, Ana Fátima Carvalho Fernandes

**Affiliations:** 2 Adjunct Professor, Departamento de Enfermagem, Centro de Ciências da Saúde, Universidade Federal de Pernambuco, Recife, PE, Brazil; 3 PhD, Associate Professor, Departamento de Enfermagem, Universidade Federal do Ceará, Fortaleza, CE, Brazil; 4 PhD, Full Professor, Departamento de Enfermagem, Universidade Federal do Ceará, Fortaleza, CE, Brazil

**Keywords:** Pregnancy, Feeding, Teaching Materials, Validation Studies, Nursing

## Abstract

**OBJECTIVE:**

to describe the validation process of an educational booklet for healthy eating in pregnancy using local and regional food.

**METHODS:**

methodological study, developed in three steps: construction of the educational booklet, validation of the educational material by judges, and by pregnant women. The validation process was conducted by 22 judges and 20 pregnant women, by convenience selection. We considered a p-value<0.85 to validate the booklet compliance and relevance, according to the six items of the instrument. As for content validation, the item-level Content Validity Index (I-CVI) was considered when a minimum score of at least 0.80 was obtained.

**RESULTS:**

five items were considered relevant by the judges. The mean I-CVI was 0.91. The pregnant women evaluated positively the booklet. The suggestions were accepted and included in the final version of the material.

**CONCLUSION:**

the booklet was validated in terms of content and relevance, and should be used by nurses for advice on healthy eating during pregnancy.

## Introduction

Printed educational materials have been used to improve knowledge, satisfaction, and adherence to treatment, as well as stimulate patients' self-care. The use of educational materials developed by health professionals as a reinforcement tool for verbal communication is recommended. The teaching material can have a positive impact on patient education and show potential for supporting patients in addressing doubts that may arise when they are not interacting with the health care provider^(^ 1 ^)^.

Nurses can use educational interventions, communicating information and evaluating educational resources developed for health education. The increasing use of educational materials enables the teaching learning process by means of interactions mediated among the speaker (nurse), patient and family (readers) and the written educational materials (discourse objects). With this, the nurse face challenges and requires clear definitions of the expected educational objectives to be achieved by the target audience^(^ 2 ^)^. The participatory approach used in the development of educational materials enables the identification of the needs of pregnant women, which indicate the inclusion of contents in the booklet that correspond to their own demands^(^ 3 ^)^.

National and international health agencies recommend that primary health care should adopt educational strategies and offer pregnant women guidance on a healthy diet and good nutrition, in order to promote health and achieve positive effects on maternal and fetal well-being^(^ 4 ^-^ 6 ^)^.

The inadequacy of the diet during pregnancy is a public health problem and increases the risk of low birth weight, deficient fetal growth, neural tube defects, maternal obesity, preeclampsia, gestational diabetes and premature birth^(^ 7 ^)^. The study that developed the Healthy Eating Index for Brazilian Pregnancy (HEIP-B) found that 62.6% of pregnant women reported diets classified as "in need of improvement," thus reinforcing the need to address nutrition education during pregnancy^(^ 8 ^)^. 

Adequate nutrition is considered important during pregnancy, and nutritional counseling beginning in the first trimester of pregnancy is a way to provide and promote behavioral change and, consequently, to reduce disease through lifestyle modifications. Moreover, it points to the use of the booklets as a way to improve nutritional counseling for patients^(^ 9 ^)^.

As new materials focused on health education are developed by professionals, these need to be evaluated to maximize their effectiveness^(^ 1 ^)^. The understanding of the procedures for content validation is important for researchers and health professionals concerned with the use of more reliable and appropriate instruments for a given population^(^ 10 ^)^. A study based on the opinion of experts and pregnant women was conducted for the construction and validation of an educational booklet for health promotion during pregnancy^(^ 3 ^)^. A similar process was applied in the evaluation of educational materials for patients undergoing orthognathic surgery^(^ 11 ^)^.

One study evaluated 59 pieces of written educational materials used for patient education. Of these, 25 were brochures, 16 were pages of plain paper (A4), and eight were booklets. The adequacy of the educational materials received low scores for content, illustration, indexing, graphics and contrast of writing style; they received high scores for level of instruction, learning, motivation and cultural adequacy. It is highlighted that written educational materials should be prepared by professionals, considering the guidelines for developing health education material for the target audience^(^ 12 ^)^.

Aiming to construct an educational booklet, the authors of this study contacted health care services and the Board of Health of Recife-PE, Brazil, to verify the existence of educational materials available for pregnant women according to the proposed theme. As a result, the absence of this type of teaching material was found.

Based on these assumptions, this study aimed to construct and validate an educational booklet about healthy diet during pregnancy, using local and regional foods, considering the participation of judges and pregnant women. The proposal to build an educational booklet included actions to promote healthy eating in primary health care, including: promote healthy eating in the health services routine; considering the life cycle; guidance and promotion of good nutritional activities that stimulate consumption of regional food; and, consideration of Brazilian cultural aspects^(^ 5 ^)^.

Considering the importance of these aspects, the study aimed to describe the process of validating an educational guide for a healthy diet with regional foods during pregnancy.

## Methods

This was a study developed by use of a methodological approach in three steps: development of the educational material for pregnant woman about healthy nutrition during pregnancy; validation of the educational material by judges; and, legitimation of the educational material by pregnant women. 

The educational materials were developed according to the recommendations for conception and efficacy of educational tools, referring to content, language, organization, layout, illustration, learning and motivation^(^ 1 ^)^. The theoretical framework upon which the material development was based was the assumptions of Motivational Interview (MI), in which two basics principles were used: to promote self-efficacy and to help address ambivalence^(^ 13 ^)^. In this way, the development of the educational material aims to motivate the pregnant woman toward healthy and adequate nutritional habits, by offering the option of choosing regional foods. 

In the development process of the educational material, a literature review was conducted by consulting the nutritional recommendations of international institutions (*European Micronutrient Recommendations Aligned, Institute of Medicine*, *World Health Organization *and *Pan American Health Organization*); Ministry of Health documents, such as *Brazilian Regional Food*; *Nutrition Guide for the Brazilian Population: Promoting Healthy Nutrition*; *Ten Steps to a Healthy Diet for Pregnant Woman*; a survey on the regional foods most consumed by pregnant woman; a survey on "Social Representation of Food Habits among Pregnant Women"^(^ 14 ^)^.

Moreover, considering the active participation of the pregnant woman, a survey on the most consumed regional foods was conducted. This approach allowed them to indicate illustrations within the primer on such foods. The survey was conducted in communities of Recife-PE-Brazil, during home visits. The results indicated considerable consumption of banana, acerola (Barbados/West Indian cherry), manioc/cassava, yams, pumpkin, sweet potatoes, and beans.

Professionals who had graduated in communication and publicity worked on the graphic design. Images were collected from the web and were later revised using *Adobe Illustrator.*

The final version of the booklet had dimensions of 148x210mm. The booklet contained eight doubled-sided pages, with a cover, back cover, table of contents, and a page for notes. Beginning on page five, there was the beginning of the organization of content on a healthy diet; foods that are allowed and to be avoided during pregnancy; the benefits of a healthy diet for the pregnant woman and her child; food preparation hygiene; and recipes using regional foods.

For evaluation of the booklet, the concept of content validity and appearance were used, namely, a judgment-based instrument that sought to measure the adequacy of assessment items with respect to the content, as well as having agreement between the judges^(^ 15 ^)^. 

A protocol for trial was designed to evaluate the content and appearance of the booklet by the judges, with six items (Table 1). The judges performed the corresponding evaluation of the agreement and relevance of each item (very irrelevant, irrelevant, relevant, very relevant). Moreover, the instrument consisted of open questions for comments and suggestions. At the end of the evaluation, the recommendations of the judges were accepted and incorporated. Subsequently, the new version of the booklet was subjected to another review process, editing and typesetting of images.

**Table 1:** Evaluation of adequacy of agreement of the educational booklet. Recife, PE, Brazil, 2013

Assessment items	p*	P^†^
1. The content covered presents relevant information on healthy eating during pregnancy	0.972	0.954
2. Texts seem clear and comprehensive	0.424	0.818
3. Illustrations used have a suitable design for adults / pregnant women	0.661	0.863
4. Illustrations presented are necessary for understanding the content	0.863	0.909
5. Illustrations and texts motivate the pregnant women / reader to understand the proposed theme	0.863	0.909
6. Applicability of the booklet in everyday clinical nursing practice	0.972	0.954

* p-value

† Binomial test

For the validation process, 22 judges participated. The sample size was calculated from the formula: n=Zα^2^.P(1-P)/e^2^, where P is the expected proportion of judges, indicating the adequacy of each item, and "e" represents the acceptable proportional difference compared to what would be expected. A confidence level of 95% was considered, indicating that at least 70 % of judges would have to rate the item as appropriate. Thus, the values used for the calculation were Zα^2^=1.96; P=0.85; e=0.15^(^ 16 ^)^. 

It is noteworthy that in the selection of judges, we considered nurses with experience in the following areas of care, teaching and/or research: women's health (pregnancy - prenatal), public health, and health education. However, the pursuit of judges occurred intentionally (by convenience), via indication of experts in the field of women's health. Thus, it was possible to identify 25 judges, and a total of 22 judges were invited to participate in the study. As research subjects, they were required to sign the Terms of Free and Informed Consent form.

Data collection was conducted from February to April of 2013. For consultation by and opinions of the judges, the following materials were given: a formal invitation, the Terms of Free and Informed Consent form, a folder with the booklet and the protocol for educational material, and a tool to characterize the profile of the professional judges.

Regarding the evaluation of content and layout by the pregnant women, they were invited to participate in the study while waiting for the prenatal clinic of the University Hospital in Recife-PE, Brazil. In a population of 35 pregnant women, a total of 20 pregnant women agreed to participate. The validation process was conducted until no new recommendations for changes were made^(^ 3 ^)^. 

For evaluation by the pregnant women, a first version of the booklet was delivered along with the guidelines for signing the Terms of Free and Informed Consent. Pregnant women were asked to handle the booklet and analyze figures and texts. For pregnant women that eventually did not understand the written text, a research assistant was instructed to perform the reading. An instrument was developed to characterize the social profile of the pregnant women (age, level of education, occupation and family income).

To review the educational booklet for pregnant women, we developed an instrument with 12 questions based on the Suitability of Assessment Materials^(^ 17 ^)^. The questions were about the women's opinion about the cover, title, subtitle, content, writing, graphics, and regarding learning motivation and cultural aspects. The women discussed the agreement and relevance of each item (very irrelevant, irrelevant, relevant, and very relevant). During the interviews, notes of comments, opinions and suggestions of the pregnant women concerning the booklet were recorded.

For the tools, data were compiled using the *Statistical Package for the Social Sciences* (SPSS), version 20.0. Statistical analysis of agreement, according to each item of the instrument, was performed using the adequacy of proportions adjustments of judges who agreed with the relevance of the educational booklet. For this, we used the Binominal test and a relevant p-value<0.85. For this analysis, the significance level (α) was set at 5%, so that p-values>0.05 indicated the proportion of judges who agreed with the appropriateness and relevance of the booklet.

The content validity index (CVI) followed three approaches: 1) I-CVI (*level content validity index*): for each one of the items, the I-CVI was calculated by the number of judges that evaluated the item as relevant or very relevant. 2) S-CVI/AVE (*scale-level content validity index, average calculation method*): the proportion of scale items rated as relevant and very relevant by each judge. 3) S-CVI (*scale-level content validity index*): mean of the proportion of items evaluated as relevant and very relevant by the judges. We considered an index >0.80 as adequate for the content validity^(^ 15 ^)^.

The study was submitted to the Board of Ethics of the Health Sciences Center of the Federal University of Pernambuco, in accordance to the recommendations by Resolution 466/2012 of the National Health Committee, and approved under the protocol number 123.140/2012.

## Results

Among the sample of 22 judges, composed of nurses, all agreed to participate in the evaluation, and returned with the material completed. Regarding the professional profile we observed 13 judges with a doctoral degree, three with PhDs in Nutrition and Public Health; five with master's degrees; three with specialization; and one post-doctoral student. Regarding their current occupation, 20 were professors and researchers; four performed care activities in prenatal consultations, two performed teaching activities. Regarding experience in writing educational material, 16 judges had such an experience. The mean time of experience in health education was 15.0 years (SD=7.9); 12.8 years in women's health (SD=9.0) and 11.2 years in public health (SD=7.9).

Regarding the social profile of the pregnant women who participated in the study, a minimum age of 19 and a maximum of 37 years old were identified, with a mean of 26 years. Regarding the level of education, four years of education was the minimum and 12 years was the maximum. The average was 8.5 years of education (SD=3.4). In terms of occupation, 60% of the women were not employed, 25% had a formal job and 15% had an informal job. Of these, 70% had a family income between one and two minimum wages (minimum wage in 2013 - BRL R$678.00).

Pregnant women positively assessed the booklet and indicated that the educational material was relevant with respect to the graphics, the complementary texts, the motivation to read, respect to the cultural aspects, as well as the clarity of writing. The suggestions of the pregnant women were included and the educational booklet was reviewed up through the final version. Some reviews of the pregnant women during the validation process were recorded:

*It draws people's attention, especially with the image of fruit *(G4).*The booklet is very colorful *(G17)*. It is attractive for those who have no information about it *(G6)*. There are some people who cannot see well, and then the cover and the colorful captions draw their attention *(G18).* It is not a tiresome reading. As the texts are short, the language is simple and direct, in a popular manner* (G16).* Many pregnant women eat everything. They only have guidance when they search for a nutritionist. The booklet is good to address that* (G2).* To read the booklet helps you to maintain eating regional foods during pregnancy *(G14)*. The booklet is important because most pregnant women do not follow the recommendations on prenatal care* (G17)*. Some cannot read and the pictures are helpful* (G10).* Those who do not know how to read, can understand the content by seeing the figures *(G17)*. That's interesting. I like the layout of the recipes showing how to cook* (G18).* It has everything within our region, such as pumpkin and cassava *(manioc, yucca) (G18).

The first version of the educational material had twelve pages. The women reported that the cover illustration aroused interest about reading the booklet. However, the judges requested improvements in the depiction of the illustrations of the father and the grandmother. The changes were accepted in order to improve the quality of the cover. The fifth page presented text and graphical displays on the concept of healthy eating. On page six, the illustrations indicated foods that are allowed and those that should be avoided during pregnancy. Page seven showed graphics and texts about the positive consequences of healthy eating.

**Figure 1:**
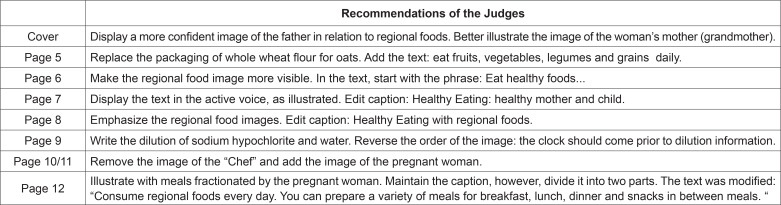
Summary of qualitative analysis of the judges' recommendations. Recife, PE, Brazil, 2013

**Figure 2:**
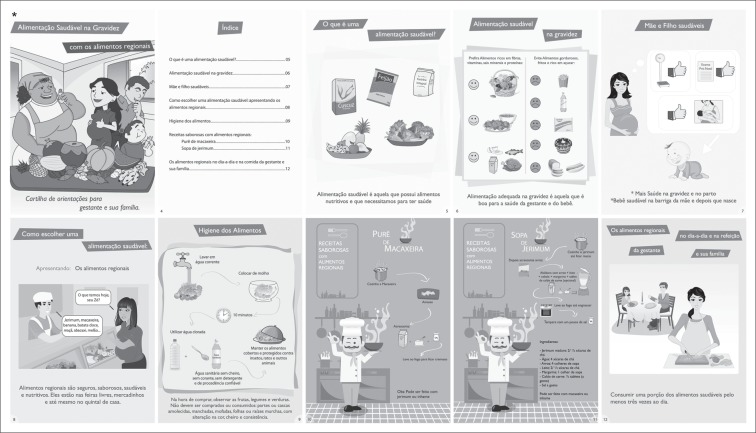
**Illustrated representation of the educational booklet presented to the judges. Recife, PE, Brazil, 2013** *Sequence of pages from left to right: Front cover. Summary, page 5 (What is healthy eating?); page 6 (Healthy Eating in Pregnancy); page 7 (Healthy Mother and Child); page 8 (How to choose a healthy diet: featuring regional foods); page 9 (Food hygiene); page 10 (Tasty recipes with regional foods: mashed manioc); page 11 (Tasty recipes with regional foods: pumpkin soup); page 12 (Regional foods in the daily life of the pregnant woman and her family)

From the eighth to the twelfth page, the illustrations and text emphasized the use of regional foods in everyday life of the pregnant woman and her family, such as banana, pumpkin, manioc/cassava, yams, sweet potatoes, coconut, and so on. It also provided recipes such as pumpkin soup and mashed cassava. The ninth page was about food preparation hygiene. However, after the evaluation by pregnant women and the judges, the final version of the booklet added a thirteenth page with meal options using regional foods (breakfast, lunch, dinner and snacks). After finishing the final version of the booklet, registration of copyright in the National Library Foundation was completed. Figure 1 presents a summary of the qualitative analysis of the recommendations made by the judges. By analyzing the booklet's folder (Figure 2), the judges gave suggestions regarding the texts and graphics. The same were accepted until the final version of the folder was adopted.

The agreement among the judges as to the adequacy and relevance of the booklet, in relation to texts, obtained a p value of 0.818. The relevance of illustrations reached a level of significance with p>0.85 and a p-value<0.05 (Table 1).

**Figure 3:**
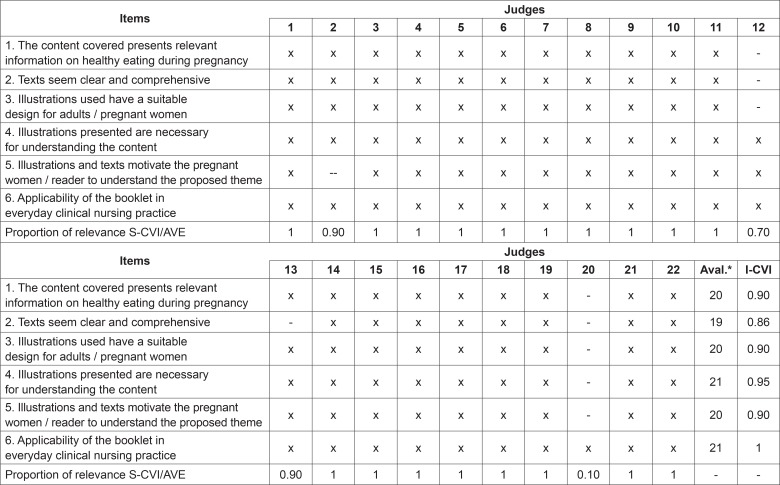
**Evaluation of the booklet regarding the six items of the instrument, according to relevance. Recife, PE, Brazil, 2013 **I-CVI Mean = 0.91 S-CVI = 0.93 *Number of very relevant or relevant reviews considered by the judges

The proportion of relevance (S-CVI/AVE) of the six items of the instrument was 100% among the 18 judges. A value of 0.93 was obtained in the S-CVI and the I-CVI mean was of 0.91. The I-CVI rated separately for each item was greater than 0.80 (Figure 3).

## Discussion

The choice for the educational booklet theme arose from reflections on the Human Right to Adequate Food, portrayed by the United Nations, in the Brazilian Law 8.080 of 1990 and in the National Policy on Food and Nutrition.

These study findings, that composed a basis for the illustrations of the most consumed regional foods by pregnant women, are a matter of concern since women presented a low intake of fruits, vegetables, roots and tubers. The decrease in consumption of these foods is part of the reality of the Brazilian population. However, it would be necessary to increase by approximately 20% the consumption of cereal, tuber and roots, and by 30% for fruits and vegetables^(^ 5 ^)^. A study conducted in Australia with 409 pregnant women showed that only 7% of pregnant women consumed vegetables, 13% fruit, and 21% alcohol^(^ 18 ^)^.

As for the concept of healthy diet, the World Health Organization includes the increased consumption of fruits, vegetables, legumes, whole grains and dried fruit, as well as a limited energy intake coming from fats and limited intake of sugars and salt (sodium)^(^ 4 ^)^. However, pregnant women should be encouraged to consume natural foods, considering the availability of regional foods, as they are sources of carbohydrates, vitamins, fiber and minerals. These foods make up the group of roots, tubers, vegetables and fruits, essential for nutritional adequacy in pregnancy^(^ 6 ^)^. Therefore, regional foods that were included in the booklet illustrations were: manioc/cassava, yam, sweet potato, pumpkin, corn, beans, coconut, banana and others.

The theoretical basis for this research enabled the authors to coordinate ideas and develop the first version of the booklet. A study of social representation regarding nutrition in pregnancy^(^ 14 ^)^ helped to clarify the key points (e.g., foods that should be avoided in pregnancy and the benefits of healthy eating). Thus, the aim was to achieve the objectives of the booklet and the needs of the target audience.

In the process of analyzing the content and the appearance of the educational material, contributions were included from pregnant women and judges who were experts in women's health, public health and health education. 

The judges provided information relevant to modification of the writing and graphics; 100% of the judges agreed on the applicability of the educational material for clinical nursing practice. The women judged this to be a relevant primer to provide guidance during prenatal visits.

Educational material effectively produced can change the reality of a population, so we must consider which information is intended and what is expected^(^ 12 ^,^ 19 ^)^. In this sense, the study suggested that pregnant women suggested the inclusion of recommendations on proper eating habits during the development of educational material^(^ 3 ^)^. Another study had nutritional counseling as a strong point of importance, in which 94.6% of the nurses agreed that guidelines on maternal nutrition during pregnancy was a widely required task in health care^(^ 9 ^)^. 

The agreement of the judges on the adequacy of the booklet content obtained a p-value of 0.972, P=0.954 and an I-CVI value of 0.90, showing this to be statistically significant information about healthy eating during pregnancy. Regarding the graphics, considering the need of presenting an appropriate design for adults, which are necessary for understanding the content and motivation of pregnant women for comprehending the proposed theme, we obtained a statistically significant P and p-value and I-CVI value >0.80.

The women included in this study assessed the texts to be very relevant (55%) and relevant (45%) concerning simplicity and clarity. The illustrations, as a complement to the texts and motivators for reading, achieved the same percentage. Women reported that the booklet attracts attention and the illustrations helped in understanding the issue. There is a consensus that health education materials should be written in a simple manner, with a lower reading level, allowing the transmission of accurate information. The illustrations should be appealing with clear communication of the purpose of the educational material^(^ 1 ^)^. Moreover, images must achieve a high level of attention and interest in reading the material, with acceptance of the population of individuals from different educational levels^(^ 20 ^)^. Judges and pregnant women identified these aspects during the assessment of the booklet under study.

The analysis of educational level among the women who participated in the review of the booklet identified a mean of 8.5 years of study. In the process of reading ability, one cannot pre-determine the level of education of the target group. It is recommended as suitable to use the range of six to eight years of study^(^ 17 ^)^. Authors showed that the level of education was of six years among the population who participated in the evaluation of educational material^(^ 20 ^)^. A study acknowledged this as a limitation of access to information for the target population and elucidated that patients with low education would likely rate the educational material with lower scores in language items^(^ 21 ^)^. It was observed that a considerable section of a booklet validation for women who underwent mastectomy was the division of patients into groups according to the level of education, i.e., primary education, secondary education and higher education^(^ 22 ^)^.

In the development process of this booklet, we considered the aspects to motivate reading and learning by pregnant women. The judges evaluated the content and the graphics as relevant to motivate the learning of the target population (p-value=0.909 and I-CVI=0.90). Studies showed that 100% of the judges who participated in the validation process of educational material agreed that content and graphics motivated patients and addressed their doubts^(^ 11 ^,^ 22 ^)^.

Health and education professionals are motivated to use the educational material as teaching tools in patient/student learning^(^ 19 ^)^. Therefore, scholars share the view on the importance of motivation to stimulate learning. Furthermore, one must consider the interaction of text and graphics in the preparation process of written health educational materials^(^ 1 ^,^ 17 ^)^.

In this sense, the educational booklet shows illustrations and text on pages 9, 10 and 11 on food hygiene and recipes for regional foods (pumpkin soup and mashed manioc). On page 13 there are suggestions for regional foods that can be used for breakfast, lunch, dinner and snacks. Such text and illustrations are intended to motivate pregnant women in food hygiene and preparation of food using accessible items from their region or communities. The women said that the recipes figured prominently and helped in their learning of other meal preparation. Corroborating the studied booklet, authors used a flipchart for a population of preschoolers, encouraging the preparation of recipes with regional foods, including recipes of beef and cashew burgers, flour with banana, pumpkin puree and cassava leaf juice^(^ 23 ^)^.

The use of Motivational Interviewing (MI) as a subsidy for the construction of the educational booklet in this study was based on two principles of the theory: self-efficacy and ambivalence. Thus, the focus for the development of texs and graphics had as a central point to help the internal motivation among pregnant woman for healthy eating and encouraging them to change, exploring the feeling of ambivalence about the importance of proper eating habits. 

A study used the MI principles to develop an educational video aimed to enhance the consumption of healthy foods during pregnancy^(^ 24 ^)^. It is important to note that the MI is an important method for the practice of health promotion, becoming a valuable tool in primary care and guidance and awareness among nurses-patients^(^ 25 ^)^.

With regard to the types of instruments used for evaluation of the booklet, the lack of a specific pattern was observed. Studies used different instruments with adaptations proposed by the authors^(^ 19 ^,^ 21 ^)^. Studies applied the *Suitability Assessment of Materials* (Doak, 1996) to review the adequacy of the educational material^(^ 12 ^,^ 20 ^,^ 22 ^)^. To validate the process judgment, some studies used a qualitative approach with the focus group technique^(^ 3 ^,^ 19 ^)^ and content analysis^(^ 2 ^)^. Other studies used the quantitative method^(^ 20 ^-^ 22 ^)^.

Quantitative analysis of the evaluation of the content and appearance of this booklet reached an S-CVI value of 0.93 and an average I-CVI value of 0.91, i.e., >0.80. In this way, the educational material, Healthy Eating with Regional Food, was considered validated. Another study showed similar results with respect to the content validity index of figures and guidance within the booklet^(^ 23 ^)^.

The relevance of the booklet, according to the applicability in everyday nurses' clinical practice, 100% of responses obtained from judges indicated its relevance and an I-CVI value of 1. The agreement among the judges about the relevance of the educational material for application in clinical practice was statistically significant (P=0.954).

Nutritional counseling and adoption of healthy eating practices should be permanent actions of the primary care professional. The nurse should include dietary guidance for pregnant women and their families during low-risk prenatal care, aiming at qualified prenatal care and satisfactory results for maternal and neonatal health^(^ 6 ^)^.

In regard to the limitations of this study, we present the use of different assessment instruments for judges and the participating pregnant women, as this did not allow the establishment of a relationship between the perceptions of the two groups. Regarding illustrated regional foods; we portrayed the ones most consumed in the Northeast region of Brazil and in the State of Pernambuco, which may limit the use of the booklet in other regions of the country.

## Conclusion

The educational material was validated for its content and relevance. The evaluation process included health professionals (judges) and pregnant women. The construction of the booklet involved scientific knowledge and teamwork, as well as engaging design and layout artists and advertising professionals. The contributions of judges and pregnant women were considered until the development of the materials' final version. The booklet is relevant, being considered as a new teaching material for health education activities, in order to motivate mothers to have a healthy diet, using regional foods.

The online version of the booklet is available to the public in the collection of the Pernambuco's E-health Network Library (Rede NUTES). However, efforts have been made to ensure the availability that the booklet in hard copy for public health institutions. A clinical trial is being developed to evaluate the effectiveness of an educational guide for behavior change with respect to the use of regional foods during pregnancy.
